# Cancer/testis antigen PIWIL2 suppresses circadian rhythms by regulating the stability and activity of BMAL1 and CLOCK

**DOI:** 10.18632/oncotarget.18973

**Published:** 2017-07-04

**Authors:** Yilu Lu, Xulei Zheng, Wei Hu, Shasha Bian, Zhiwei Zhang, Dachang Tao, Yunqiang Liu, Yongxin Ma

**Affiliations:** ^1^ Department of Medical Genetics, State Key Laboratory of Biotherapy, West China Hospital, Sichuan University, Chengdu, Sichuan 610041, China

**Keywords:** circadian clock, PIWIL2, GSK3β, ubiquitination

## Abstract

Circadian rhythms are regulated by transcriptional and post-translational feedback loops generated by appropriate functions of clock proteins. Rhythmic degradation of the circadian clock proteins is critical for maintenance of the circadian oscillations. Notably, circadian clock does not work during spermatogenesis and can be disrupted in tumors. However, the underlying mechanism that suppresses circadian rhythms in germ cells and cancer cells remains largely unknown. Here we report that the cancer/testis antigen PIWIL2 can repress circadian rhythms both in the testis and cancer cells. By facilitating SRC binding with PI3K, PIWIL2 activates the PI3K-AKT pathway to phosphorylate and deactivate GSK3β, suppressing GSK3β-induced phosphorylation and degradation of circadian protein BMAL1 and CLOCK. Meanwhile, PIWIL2 can bind with E-Box sequences associated with the BMAL1/CLOCK complex to negatively regulate the transcriptional activation activity of promoters of clock-controlled genes. Taken together, our results first described a function for the germline-specific protein PIWIL2 in regulation of the circadian clock, providing a molecular link between spermatogenesis as well as tumorigenesis to the dysfunction of circadian rhythms.

## INTRODUCTION

Circadian rhythms show universally a 24-h oscillation pattern in almost all species from prokaryotes to humans. In mammals, the circadian network is generated and maintained via tightly regulated transcriptional-translational feedback loops [1–3]. The core heterodimer, composed of BMAL1 and CLOCK proteins, induces the expression of circadian output genes, as well as the negative components of the circadian loops by binding to the E-Box elements on their promoters. The most studied repressors are Period (Per) and Cryptochrome (Cry) proteins which bind to and inhibit BMAL1/CLOCK [1]. The other negative loop is mediated by the nuclear receptor Rev-erbα which directly represses *Bmal1* gene expression [4–6].

Evidence has demonstrated that various core clock components are subjected to post-translational modifications (PTMs) that participate in controlling the activation and the repression of circadian transcription [7]. In recent studies, glycogen synthase kinase 3 beta (GSK3β) has been identified as a critical regulator of stability and activity of circadian proteins, including Bmal1 [8], Clock [9], Period [10], Cryptochrome [11] and Rev-erba [12, 13]. Generally, GSK3β phosphorylates circadian proteins and thus regulates their ubiquitination and proteasome-dependent degradation. Yet, the signaling pathways controlling the plasticity of the circadian system have not been deciphered.

Circadian clocks are present in almost all the tissues in mammals. The master clock is located in the hypothalamic suprachiasmatic nucleus (SCN), while peripheral clocks are present in other mammalian tissues, such as liver, heart, lung and kidney, where they maintain circadian rhythms and regulate tissue-specific gene expression. However, recent studies showed mammal male germ cells devoid of circadian rhythm [14, 15], while disruption of circadian rhythms has been reported in various forms of human cancers [16, 17]. Considering the similarity between germ cells and cancer cells, we propose a hypothesis that the dysfunction of circadian clock in male germ cells and cancer cells may be attributed to certain proteins that express specifically in the testis and cancer cells, namely, cancer/testis antigens (CTAs).

PIWIL2, aka HILI in human, is a novel CTA protein that plays essential roles in spermatogenesis and embryogenesis [18–20]. The expression of PIWIL2 in early benign hyperplasia and precancer stem cells suggests that PIWIL2 may play important roles in tumorigenesis while the underlying mechanism remains largely unclear [21, 22]. Our previous researches had first indicated that PIWIL2 functions to induce SRC kinase to phosphorylate and activate the STAT3 pathway to prevent expression of P53, resulting in apoptosis inhibition [23]. Besides, we have provided evidence showing that PIWIL2 has the capacity to regulate ubiquitination and proteasome-dependent degradation of TGF-β receptor [24, 25] and intermediate filament Keratin 8 [26].

Here we present that PIWIL2 represses circadian rhythms both in the testis and cancer cells. Evidences suggested that PIWIL2 facilitates a SRC-PI3K-AKT pathway repressing the activity of GSK3β to protect circadian protein BMAL1 and CLOCK from ubiquitination and degradation. Meanwhile, PIWIL2 can bind with E-Box sequences associated with the BMAL1/CLOCK complex to negatively regulate the transcriptional activation activity of CCG (clock controlled genes) promoters. Our work suggests a novel mechanism to suppress circadian cycling in spermatogenesis and tumorigenesis.

## RESULTS

### Knockdown of PIWIL2 decreases BMAL1 and CLOCK expression

**Figure 1:**
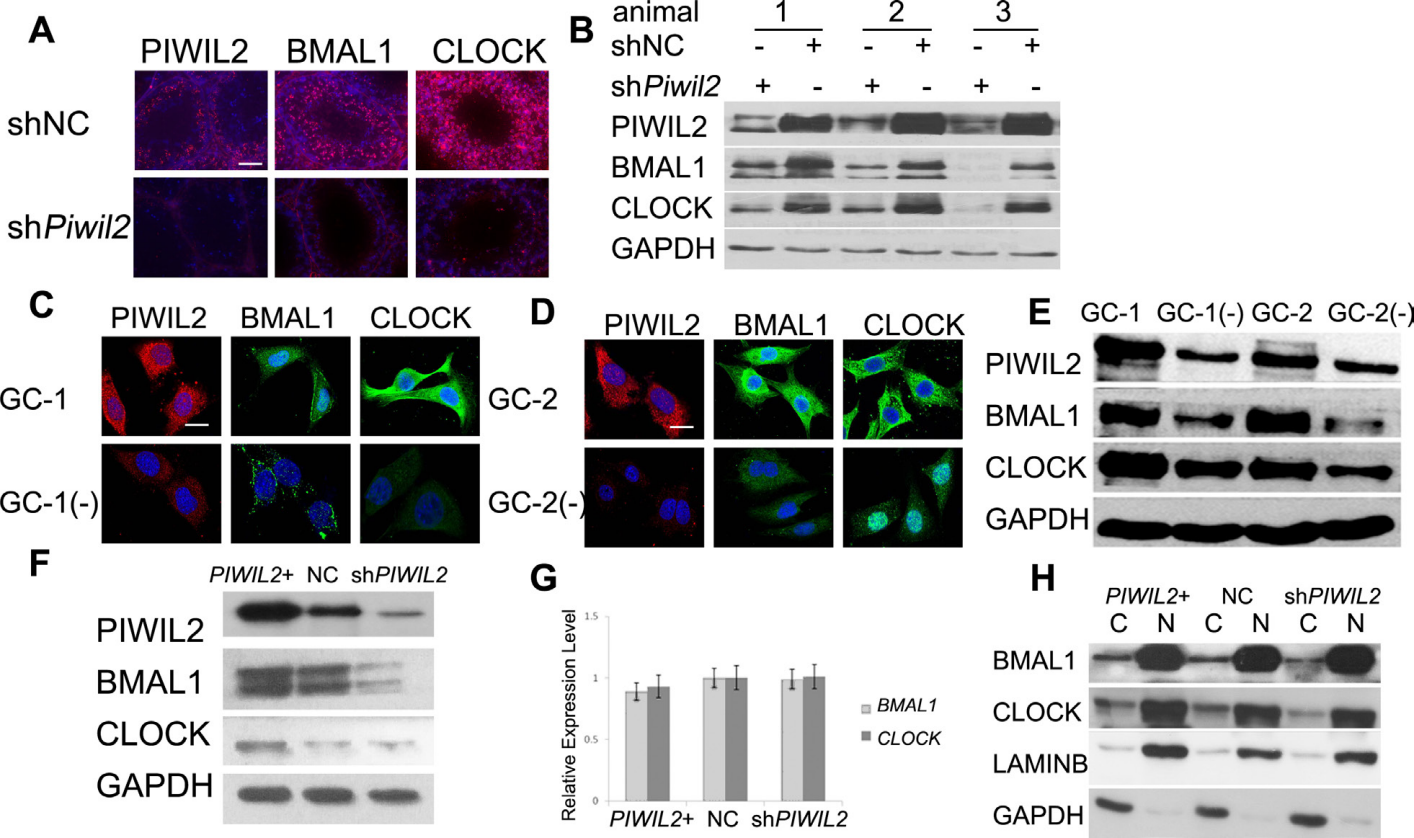
PIWIL2 promotes expression of BMAL1 and CLOCK (**A**) Fluorescent immunohistochemistry (IHC) showed expression of PIWIL2, BMAL1 and CLOCK in two separated testis samples of the same mouse, respectively injected with *Piwil2*-specific shRNA vectors (sh*Piwil2*) and empty vectors (shNC). Scale bar corresponds to 100 mm. (**B**) Western blotting showing expression of BMAL1 and CLOCK in injected testis samples from three mice. (**C**, **D**) Fluorescent staining of BMAL1 and CLOCK decreased in *Piwil2*-knocked-down germ cells. GC-1(−), stable *Piwil2*-knocked-down GC-1 cell line. GC-2(−), stable *Piwil2*-knocked-down GC-2 cell line. Scale bar corresponds to 10 mm. (**E**) Western Blotting showed that expression of BMAL1 and CLOCK decreased in *Piwil2* knocked-down germ cells. (**F**) PIWIL2 promotes expression of BMAL1 and CLOCK in HeLa cell. *PIWIL2*+, *PIWIL2* overexpressed HeLa cells; NC, HeLa cells transfected with empty vectors; sh*PIWIL2*, *PIWIL2* silenced HeLa cell. (**G**) No significant change on mRNA level of *BMAL1* and *CLOCK* in *PIWIL2* overexpressed or knocked-down HeLa cells. (**H**) cytosolic (C)/nuclear (N) fractionation assay. Lamin B1 and GAPDH were employed as internal controls.

To investigate the effect of PIWIL2 on circadian protein such as BMAL1 and CLOCK, shRNA expression vectors were injected into each testis of the same mouse. Fluorescent IHC and Western bloting experiments were performed and showed that injection of mouse *Piwil2*-specific shRNA vectors significantly decreased expression of BMAL1 and CLOCK (by 67% and 75%, respectively) in mouse testis compared with mock injection of nonspecific shRNA vectors (Figure 1A, 1B).

Then we established stable lines of *Piwil2*-knocked-down GC-1 (mouse spermatogonia) and GC-2 (mouse spermatocyte) cells, detailed in Supplementary Figure 1. Western bloting and immunofluorescence results showed that expression of BMAL1 and CLOCK decreased in *Piwil2*-knocked-down GC-1 (by 51% and 71%, respetively) and GC-2 cells (by 60% and 46%, respetively) (Figure 1C–1E).

Previous studies indicated that PIWIL2 is expressed not only in germ cells but also in cancer cells, playing important roles in tumorigenesis [21, 23]. So we next examined whether PIWIL2 regulates circadian proteins in tumor cells. Western bloting results showed that expression of BMAL1 and CLOCK decreased in *PIWIL2*-knocked-down HeLa cells (Figure 1F). However, mRNA levels of BMAL1 and CLOCK can not be altered by overexpression or knockdown of PIWIL2 (Figure 1G). Interestingly, The cytosolic/ nuclear fractionation assays further showed that knockdown of *PIWIL2* down-regulates BMAL1 and CLOCK mostly in the cytoplasm (Figure 1H also showed in Supplementary Figure 2). Above results suggested that PIWIL2 plays a role in regulating circadian proteins in mutiple tissues and organisms.

### PIWIL2 interacts with BMAL1 and CLOCK via PIWI domain

**Figure 2:**
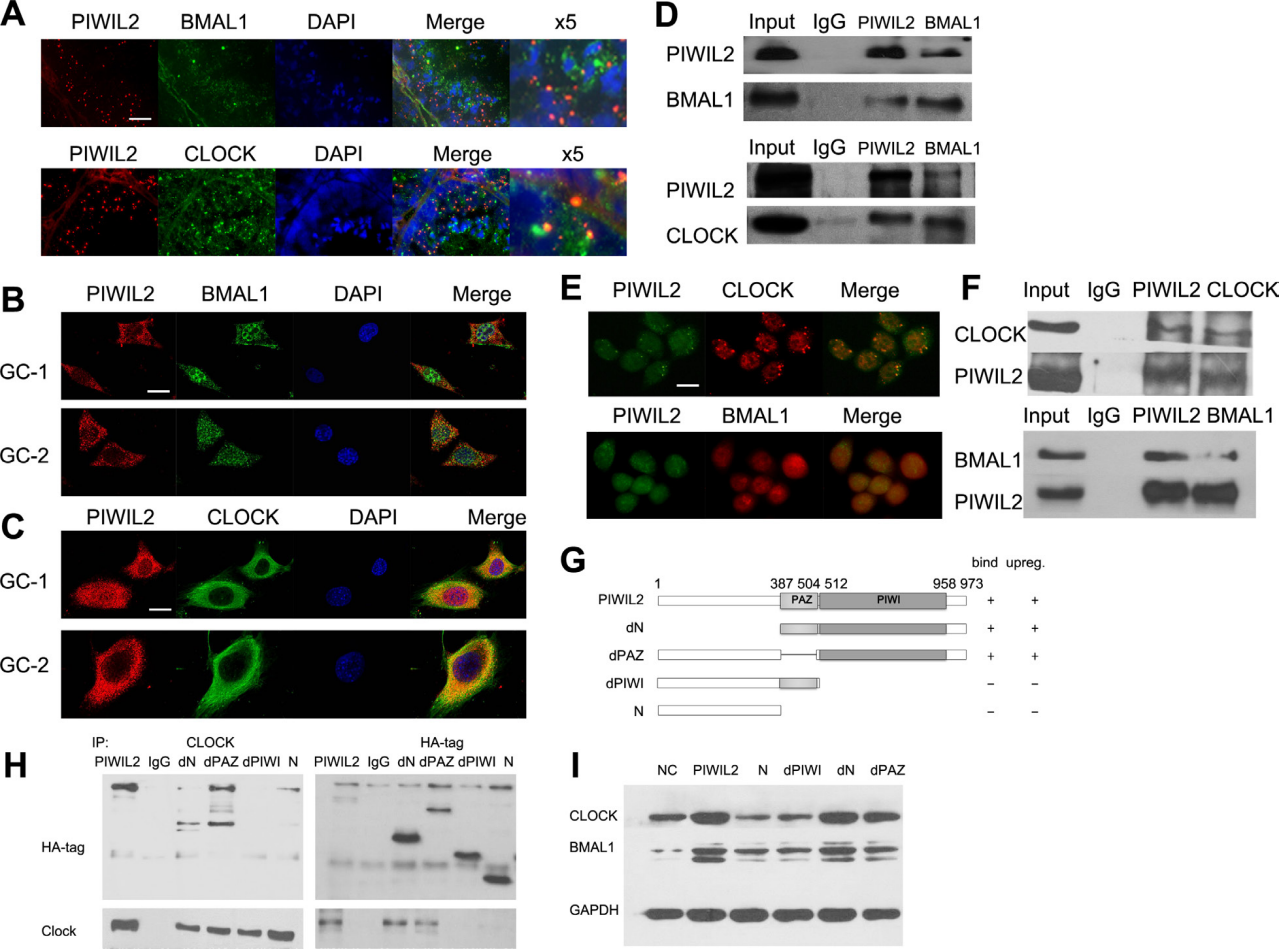
PIWIL2 interacts with BMAL1 and CLOCK (**A**) Fluorescent IHC staining of PIWIL2 overlapped with BMAL1 and CLOCK in mouse testis. X5, Part of the images was shown at a 500% higher resolution. Scale bar corresponds to 50 mm. (**B**) PIWIL2 and BMAL1 were co-localized in GC-1 and GC-2 cells. Scale bar corresponds to 10 mm. (**C**) PIWIL2 and CLOCK were co-localized in GC-1 and GC-2 cells. Scale bar corresponds to 10 mm. (**D**) Immunoprecipitation showed interactions between PIWIL2 and BMAL1/CLOCK in mouse testis. (**E**) PIWIL2 and BMAL1/CLOCK were co-localized in HeLa cells. Scale bar corresponds to 15 mm. (**F**) Endogenous interactions between PIWIL2 and BMAL1/CLOCK in HeLa cells. Input, protein sample without immunoprecipitation. (**G**) Schematic of different PIWIL2 mutants, showing their capability of binding with (**H**) and/or upregulating (**I**) circadian proteins.

Confocal immunofluorescence showed that PIWIL2 staining was overlapped with BMAL1 and/or CLOCK in mouse testis (Figure 2A), suggesting that PIWIL2 can interact with circadian protein like BMAL1 and CLOCK. Further experiments also showed that PIWIL2 is co-localized with BMAL1 and CLOCK both in mouse spermatogonia (Figure 2B) and in mouse spermatocyte cells (Figure 2C). Immunoprecipitation assay (IP) was performed to verify this hypothesis. And the result showed that PIWIL2 can directly bind with BMAL1 and CLOCK in mouse testis (Figure 2D).

As we have shown that PIWIL2 regulates circadian proteins in cancer cells as well as mammal germ cells. Interation between PIWIL2 and circadian proteins were also examined in HeLa cells. IP and immunofluorescence experiments showed that PIWIL2 can bind with CLOCK and/or BMAL1 in HeLa cells (Figure 2E, 2F).

We further deployed a series of PIWIL2 deletion mutants to identify the functional domain(s) required for interaction with circadian proteins (Figure 2G). Two PIWIL2 mutants, one harbored a deletion of PIWI conserved domain while the other lacks both PAZ and PIWI domains, failed to effectively bind with CLOCK protein, while other mutants retained the ability (Figure 2H). Meanwhile, the PIWI-deleted mutants failed to increase expression of BMAL1 and CLOCK, confirming the potential role of PIWI domain of PIWIL2 (Figure 2I).

### PIWIL2 suppresses GSK3β induced phosphorylation and ubiquitination of BMAL1 and CLOCK proteins

**Figure 3:**
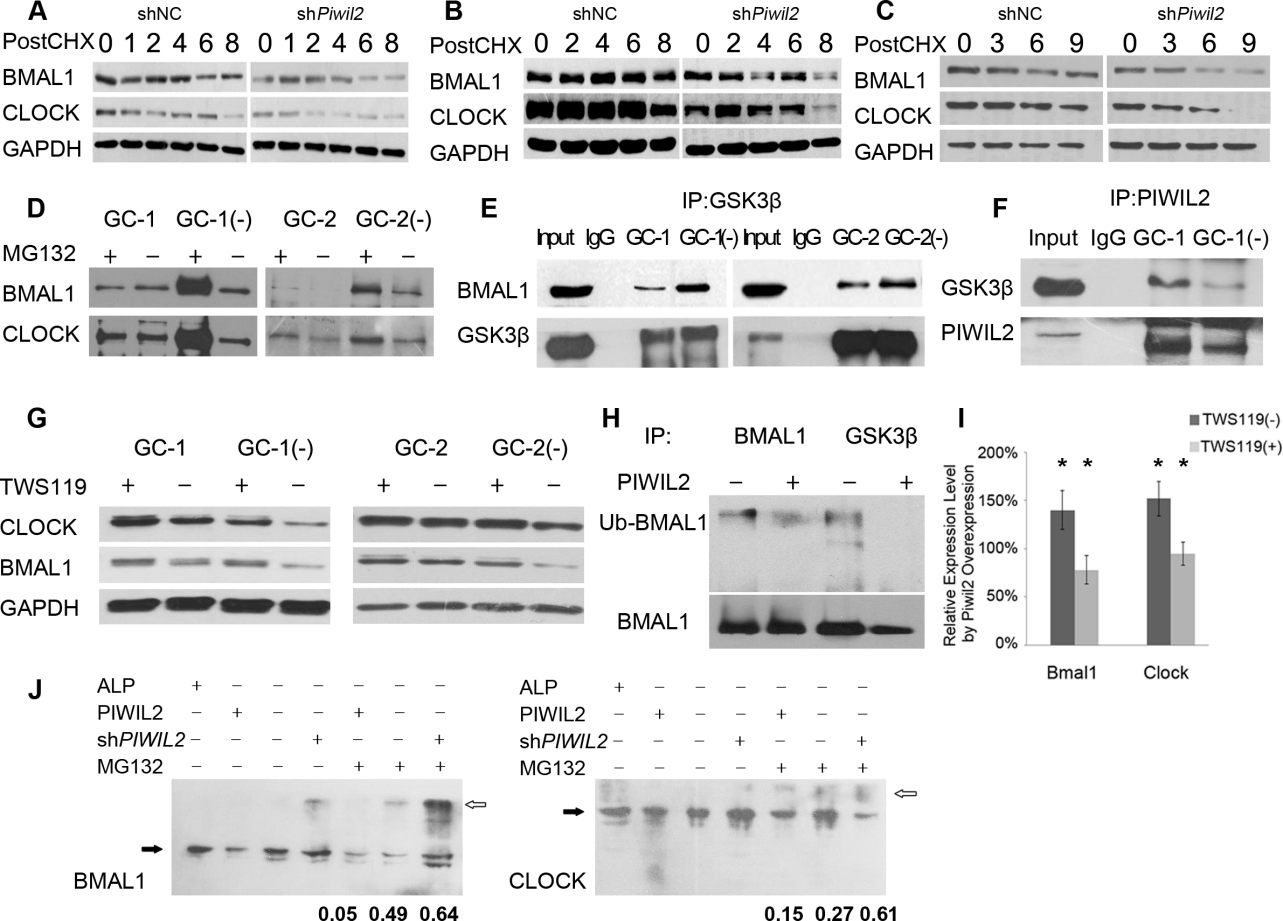
PIWIL2 blocks GSK3β induced ubiquitination and degradation of BMAL1 and CLOCK (**A**–**C**) Knockdown of *Piwil2* decreases the stability of BMAL1 and CLOCK in GC-1 (A), GC-2 (B) and HeLa (C) cell lines. Cells were treated with cycloheximide (CHX) at 5 mg/ml for indicated time (hours). (**D**) More BMAL1 and CLOCK proteins were ubiquintinated in *Piwil2* knocked-down GC-1 and GC-2 cells. (**E**) Knockdown of *Piwil2* promotes the interaction between GSK3β and BMAL1/CLOCK in GC-1 and GC-2 cells. Input, total protein as positive control. (**F**) PIWIL2 binds with GSK3β in a dose-dependent manner. (**G**) Treatment with TWS119 recovers expression of BMAL1 and CLOCK in *Piwil2* knocked-down GC-1 and GC-2 cells. (**H**) Overexpression of *PIWIL2* decreased the interaction between GSK3β and BMAL1, as well as the ubiquitination level of BMAL1. (**I**) Treatment of TWS119 abates the upregulation of BMAL1 and CLOCK by PIWIL2. Data are represented as mean ± SEM. (**J**) Phos-tag SDS-PAGE results showed that PIWIL2 promotes phosphorylation of BMAL1 and CLOCK. White arrows indicate phosphorylated proteins while black arrows indicate unphosphorylatesd proteins. The numbers indicate phosphorylation ratios of BMAL and CLOCK (phosphorylated vs total protein, *n* = 3). ALP, alkaline phosphatase.

Circadian rhythms are known to be regulated by transcriptional and post-transcriptional feedback loops involving a set of circadian proteins. Post-translational modifications, *e.g*. ubiquitination and phosphorylation contribute signficantly to stability of these proteins, which is a key regulatory step to the plasticity of the circadian systems [8]. First we examined whether PIWIL2 promotes the stability of circadian proteins. Germ cells (GC-1 and GC-2) and cancer cells (HeLa) were treated with cycloheximide (CHX) to inhibit protein biosynthesis and harvested for western bloting analysis. All three cell lines with *PIWIL2* knocked-down showed higher degradation level of BMAL1 and CLOCK in contrast to control cells (Figure 3A–3C), suggesting that PIWIL2 promotes the stability of BMAL1 and CLOCK.

Then we treated GC-1 and GC-2 cells with proteasome inhibitor MG132, and immunoprecipitated the cell lysis with anti-Ubiquitin antibody. The result showed that significantly more BMAL1 and CLOCK protein are ubiquitinated and then degradated by proteasome in *PIWIL2* knocked-down cells (Figure 3D).

Previous studies suggested that GSK3β plays important roles in mammalian circadian clock and can induce phosphorylation and degradation of BMAL1 and CLOCK [8, 9, 13]. However, it is not clear how GSK3β-induced circadian rhythm regulation works in male germ cells. So we would like to know whether PIWIL2 blocks GSK3β induced phosphorylation and ubiquitination that decrease the stability of circadian protein. Immunoprecipitation results showed that significantly more BMAL1 protein were pulldowned by anti-GSK3β in *PIWIL2* knocked-down germ cells (Figure 3E). Meanwhile, less GSK3β binds with PIWIL2 in *PIWIL2* knocked-down germ cells, suggesting that PIWIL2 may play as a competitive inhibitor to the interaction between GSK3β and circadian proteins (Figure 3F). Then we treated cells with GSK3β inhibitor TWS119 and western blot experiments showed that knockdown of *PIWIL2* no longer decreases protein level of BMAL1 and CLOCK in absence of GSK3β (Figure 3G).

Also, our further study on HeLa cancer cells showed consistent results that PIWIL2 suppresses the interaction between GSK3β and BMAL1, leading to the failure of ubiquitination and degradation of the latter protein (Figure 3H, 3I). To further prove the impact of PIWIL2, Phos-tag SDS-PAGE gels were deployed to separate phosphorylated circadian proteins. The results showed that PIWIL2 inhibits phosphorylation of BMAL1 and CLOCK (Figure 3J).

### PIWIL2 enhances a SRC-PI3K-AKT pathway to inhibit GSK3β activity

**Figure 4:**
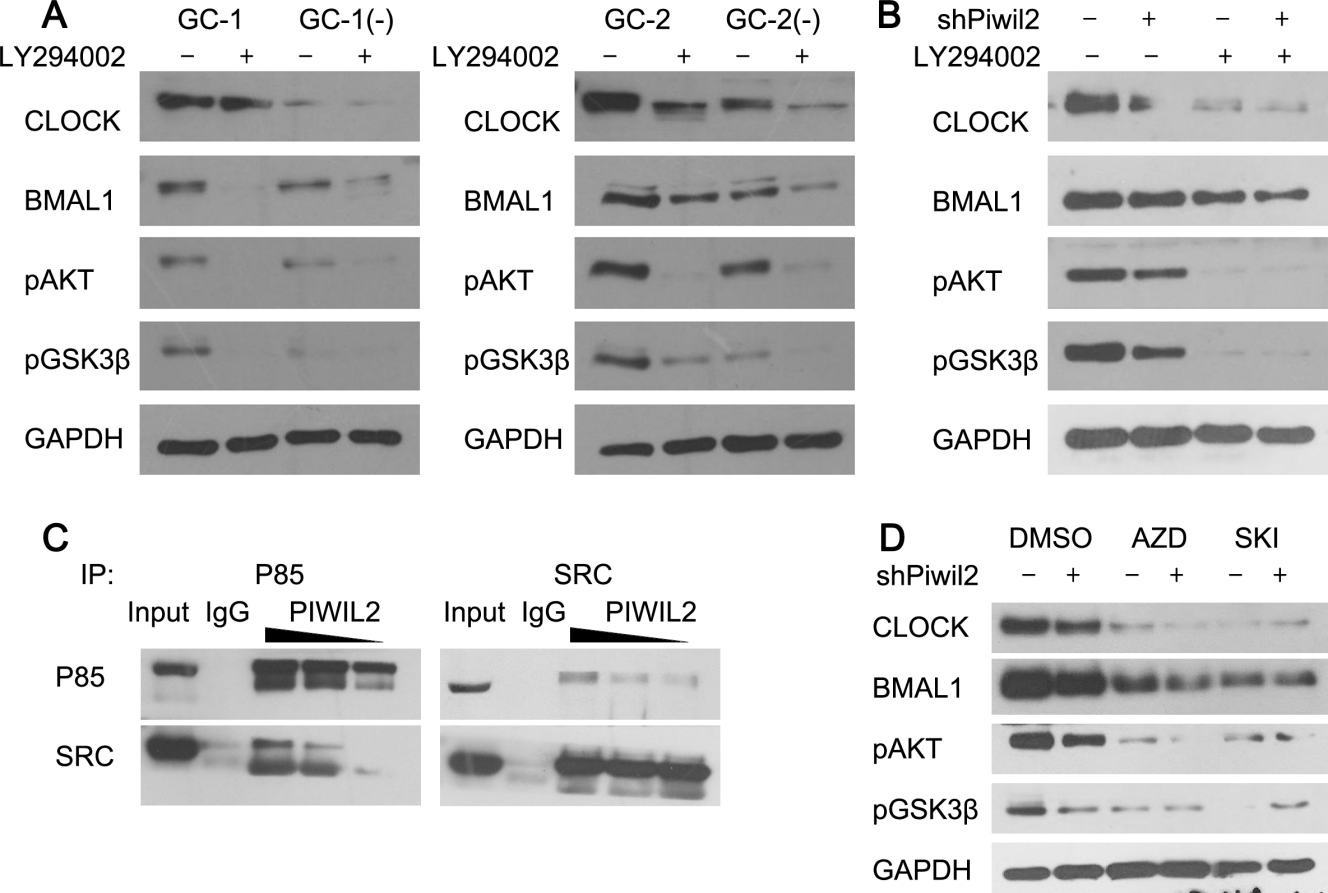
PIWIL2 facilitates SRC binding with PI3K to activate PI3K-AKT pathway (**A**) Knockdown of *Piwil2* significantly decreased the phosphorylation of AKT(S473) and GSK3β(S9) in GC-1 and GC-2 cells. And this effect can be attenuated by PI3K inhibitor LY294002. (**B**) Treatment of LY294002 weakens PIWIL2-dependent stability of BMAL1 and CLOCK in Hela cells. (**C**) PIWIL2 increases the binding between SRC and p85 subunit of PI3K. TP, total protein as positive control. (**D**) Treatment of SRC inhibitors weaken PIWIL2-dependent stability of BMAL1 and CLOCK in Hela cells. AZD, Saracatinib (AZD0530); SKI, Bosutinib (SKI-606).

As GSK3β is a kinase normally active in unstimulated cells and can be negatively regulated by phosphorylation at Ser9 by AKT and other kinases [27, 28], we next examined whether PIWIL2 not only blocks GSK3β from interacting with circadian proteins but also suppresses its activity. Our results showed that PIWIL2 can significantly up-regulate phosphorylation level at Ser9 of GSK3β in germ cells (Figure 4A) and cancer cells (Figure 4B). And this effect can be abolished by LY294002, an inhibitor of PI3K-AKT signaling pathway.

Our previous study has shown that PIWIL2 can promote the phosphorylation of STAT3 by tyrosine-protein kinase SRC [23]. And SRC is known to control PI3k-induced AKT phosphorylation, and regulate mutiple biochemical processes involving substrates of PI3K-AKT pathway, *e.g*. GSK3β [29–31]. So we assumed that PIWIL2 may facilitate SRC-induced kinase activity of PI3K, and in turn phosphorylate AKT and GSK3β. By using immunoprecipitation method, we observed that overexpression of *PIWIL2* increases the interaction between SRC and p85 regulatory subunit of PI3K, while knockdown of *PIWIL2* weakens this interaction (Figure 4C). Furthermore, when SRC inhitors were introduced, expression level of BMAL1 and CLOCK, as well as Ser9-phosphorylated GSK3β were significantly decreased. And knockdown of *PIWIL2* can not recover the effect of SRC inhibitors, suggesting that PIWIL2 enhances stability of circadian protein BMAL1 and CLOCK in a SRC-dependent manner (Figure 4D).

### PIWIL2 negatively regulates transcriptional activation activity of E-BOX elements

**Figure 5:**
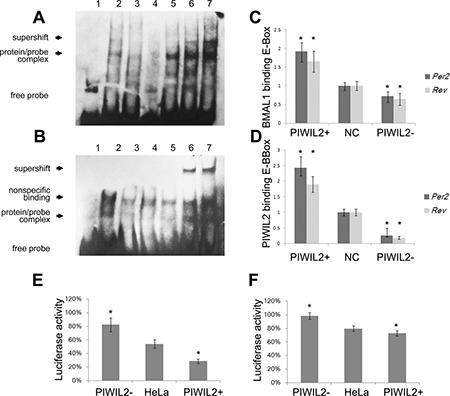
PIWIL2 enhances transcriptional activation activity of E-BOX elements (**A**, **B**) EMSA experiments showed that PIWIL2 can direct bind with probes containing sequence of E-Box regions on promoter of Per2 (A) and Rev-Erbα (B). Lanes: 1, negative control with no protein; 2-7, 2μl HeLa nuclear extract; 3, mutant probe; 4, cold competition with unlabelled DNA; 5, cold competition with mutant unlabelled DNA; 6, supershifted with 1μg anti-BMAL1 antibody; 7, supershifted with 1μg anti-PIWIL2 antibody. (**C**, **D**) Relative binding levels to E-BOX elements of BMAL1 (C) and PIWIL2 (D) were examined by ChIP–qPCR assay. Fold differences were calculated by 2^−ΔΔCt^. (**E**, **F**) Transcriptional activation activity of E-BOX elements on promoter of Per2 (E) and Rev-Erbα (F) were tested using dual Luciferase reporter assay. Data are represented as mean ± SEM. *P < 0.05.

As we have provided evidences that PIWIL2 enhances the stability of BMAL1 and CLOCK, it is a matter of interest to investigate whether PIWIL2 regulates clock-controlled genes (CCG) by employing BMAL1/CLOCK complex to the E-Box region on CCG promoters. By using electrophoretic mobility shift assay (EMSA), we observed that PIWIL2 protein from HeLa cell lysis can bind with Biotin-labeled probes containing sequence of E-Box regions on promoter of *PER2* and *Rev-Erbα*, respectively (Figure 5A, 5B). As PIWIL2 does not possess a DNA-binding motif such as bHLH, it may not bind to the E-boxes directly, rather than via binding to CLOCK and BMAL1.

Then we performed chromatin immunoprecipitation (ChIP) experiments to explore whether PIWIL2 binds with the E-Box region on CCG promoters in HeLa cells. Quantifying with real-time PCR, we observed that PIWIL2 can bind with the E-Box regions on promoter of *PER2* and *Rev-Erbα* in HeLa cells, and significantly enhance the interaction between BMAL1 and E-BOX elements (Figure 5C, 5D).

Additionally, we constructed two reporter gene vectors with E-Box regions on promoter of *PER2* and *Rev-Erbα*, respectively. Dual Luciferase reporter gene assay showed that both E-Box sequences were negative transcriptional elements in HeLa cells. Notably, when *PIWIL2* was overexpressed, relative luciferase activity was significantly decreased; and vice versa (Figure 5E, 5F). Above results suggested that PIWIL2 negatively regulates the transcriptional activation activity of E-BOX element on promoter regions of *PER2* and *Rev-Erbα*.

### PIWIL2 suppresses circadian clock by controlling BMAL1 and CLOCK

**Figure 6:**
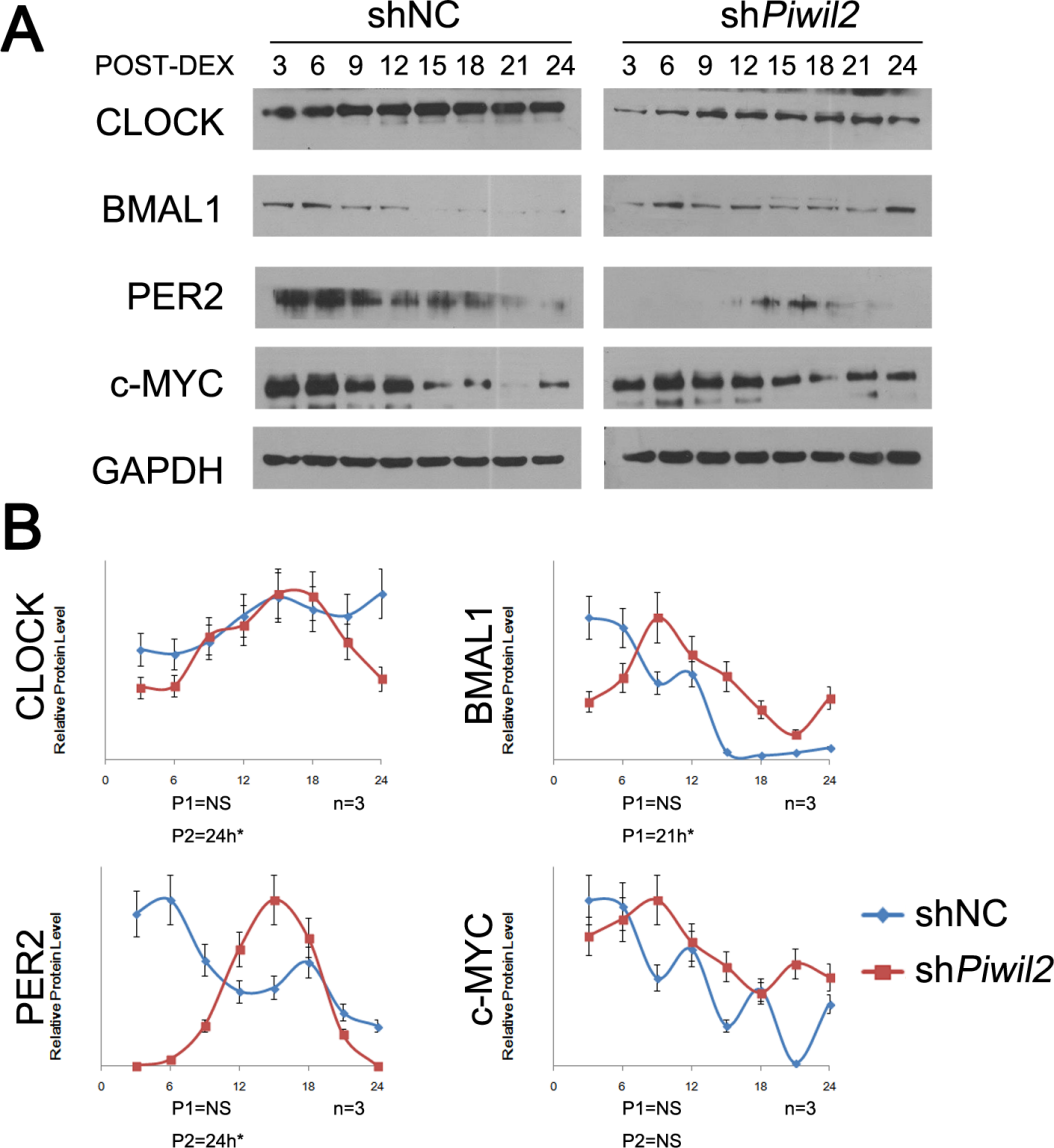
Knockdown of *PIWIL2* restores circadian rhythms HeLa cells transfected with shNC or sh*Piwil2* were synchronized by 2 hour treatment with 100nM dexamethasone (DEX). (**A**) Total lysates were prepared at indicated times post synchronization for Western analysis. Data are represented as mean ± SEM. (**B**) The intensity of bands were quantified using ImageJ software, and the data were normalized to an arbitrary value of 1.00 based on the peak of expression level. The rhythm periods of HeLa cells transfected with shNC (P1) or sh*Piwil2* (P2) were determined using JTK_Cycle software. NS, no significant rhythm. *P < 0.05.

The regulation of BMAL1 and CLOCK by PIWIL2 may impact on the cyclic expression of clock-controlled genes. We thereby examined whether PIWIL2 affects circadian rhythms. WT and *PIWIL2* knocked-down HeLa cells were synchronized by dexamethasone, and harvested for western blotting. The protein levels were quantified using ImageJ software, then normalized and subjected to rhythmic test using JTK_Cycle software. The results showed that the expression of BMAL1, CLOCK and PER2 in WT HeLa cells do not exhibit an overt circadian rhythm, consistent with previous reports [32, 33]. Howerver, *PIWIL2* knocked-down HeLa cells showed a roughly 24-hrs’ oscillation of circadian protein expression after dexamethasone treatment (Figure 6).

Notably, *c-MYC*, an oncogene plays as critical regulator of the cell cycle through downregulation of p21 and activation of CyclinD1, is regulated by the circadian system via multiple E-box sequences on its promoter [34]. Western blot experiment suggested that c-MYC protein level may exhibit circadian oscillation in *PIWIL2* knocked-down HeLa cells. However, the rhythmic expression of c-MYC was not significant (Figure 6).

Together, these results indicated that lack of PIWIL2 can recover circadian oscillation of both circadian genes and clock-controlled genes in cancer cells devoid of circadian rhythm, suggesting that PIWIL2 may suppress circadian rhythms by regulating post-translational modifications of BMAL1 and CLOCK.

## DISCUSSION

Circadian rhythms are regulated by transcriptional and post-translational feedback loops generated by appropriate functions of clock proteins. Controlled degradation of the negative-feedback loop proteins is critical for the maintenance of circadian oscillations [6]. Our finding that PIWIL2 suppress GSK3β-induced phosphorylation to regulate the stability of circadian protein BMAL1 and CLOCK, first implicates PIWIL2 as a novel regulator of the circadian network.

Previous researches including ours indicated that PIWIL2 belongs to the category of cancer/testis antigens (CTA) that expressed in human tumors but not in normal adult tissues except for testis [21, 23, 26, 35]. Notably, it has been reported that no circadian rhythm is observed in male germ cells [14, 15], while disruption of circadian rhythms is associated with various forms of cancer in humans [16, 17]. This similarity implies that the dysfunction of circadian clock in male germ cells and cancer cells may be attributed to regulation by certain CTAs, which is supported by recent finding that a cancer/testis antigen PASD1 can suppress the circadian clock [36].

Our previous study indicated that PIWIL2 can interact with SRC kinase to promote the STAT3 pathway [23]. And now we provided evidences suggesting that PIWIL2 facilitates SRC binding with PI3K, activates PI3K-AKT pathway, and phosphorylates GSK3β to repress its kinase activity. Thus, with GSK3β phosphorylated and inactivated, circadian protein BMAL1 and CLOCK can not be properly phosphorylated, ubiquitinated and then degradated by proteasome. Furthermore, we found that the effect of PIWIL2 on circadian clock is not limited to repress the regulatory kinase GSK3β, but also can bind to certain E-Box sequences associated with circadian proteins to negatively regulate the transcriptional activation activity of *PER2* and *Rev-Erbα* promoters. As PIWIL2 does not possess a DNA-binding motif such as bHLH, it may bind to the E-boxes via binding to BMAL1 and CLOCK. This finding also extends the nuclear function of PIWIL2.

Interestingly, by deploying Dual Luciferase reporter gene assay, it is implied that E-Box sequences from promoters of *PER2* and *Rev-Erb*α may play as negative cis-acting elements in HeLa cells, rather than their canonical function as enhancers. As previous report suggested, the CLOCK/BMAL1 complex can suppress the activity of certain CCG promoters upon its interaction with other transcription factors [37]. Also, it is suggested that GSK3β-induced phosphorylation may increase the activity of BMAL1 and CLOCK while decrease their stability [8, 9, 38]. These studies are consistent with our finding that PIWIL2 protects circadian core protein BMAL1 and CLOCK from degradation but may reverse their transcriptional activation activity on *PER2* and *Rev-Erbα*, leading to accumulation of the circadian core proteins BMAL1 and CLOCK and disruption of the negative-feedback loops. This hypothesis is supported by the fact that knockdown of PIWIL2 can recover the disrupted circadian system in HeLa cancer cells to a certain extent.

**Figure 7:**
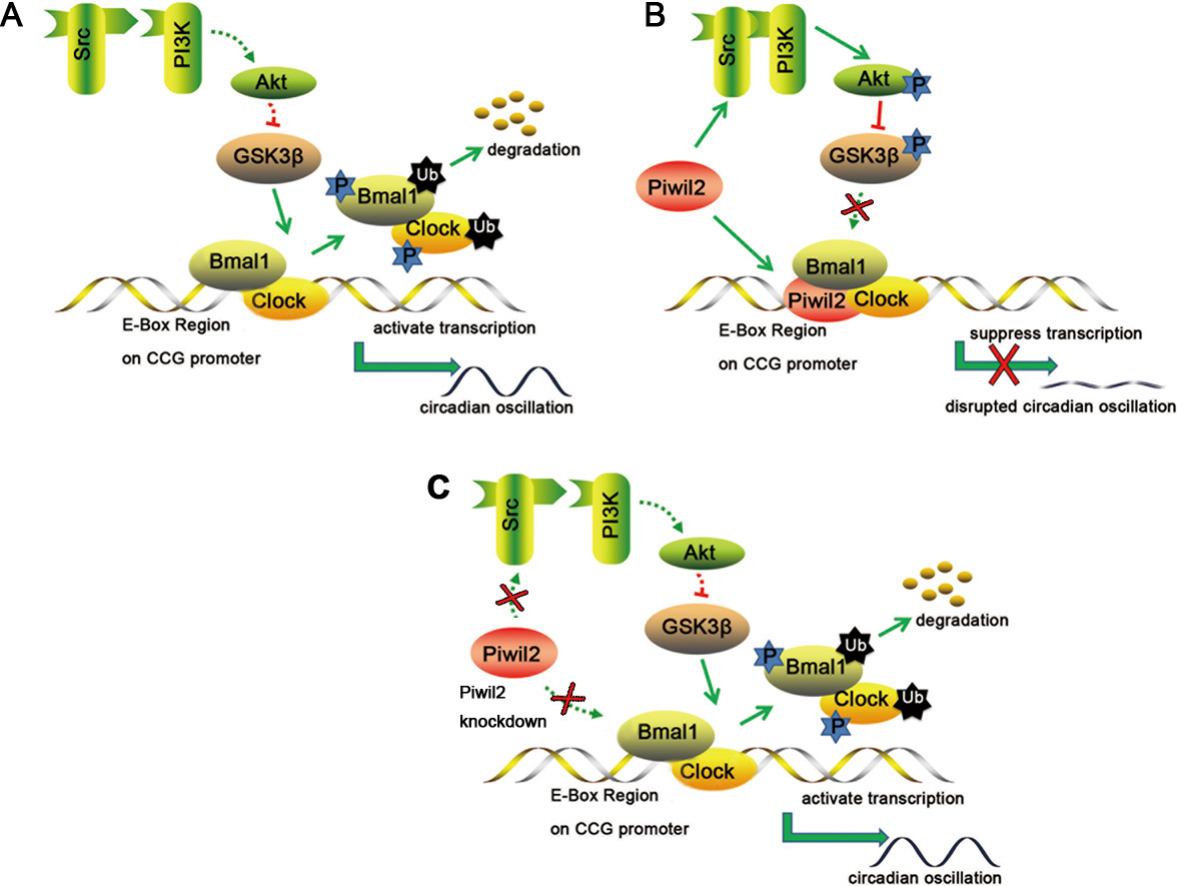
A schematic model for the involvement of PIWIL2 in circadian clock (**A**) In normal somatic cells, GSK3β phosphorylates BMAL1 and CLOCK, induces ubiquitination and degradation of these proteins, and helps to maintain the circadian feedback loops. (**B**) In germ cells or cancer cells, PIWIL2 facilitates SRC binding with PI3K to phosphorylate and deactivate GSK3β, leading to accumulation of BMAL1 and CLOCK. Meanwhile, PIWIL2 can also repress the transcriptional activation activity of E-Box enhancer and disrupt the circadian oscillation. (**C**) In germ cells or cancer cells, knockdown of PIWIL2 leads to degradation of BMAL1 and CLOCK, and restores circadian rhythm.

In summary, our present study provided evidences showing that PIWIL2 may regulate circadian system in mutiple tissues and organisms via at least two pathways: 1) To initiate a SRC/PI3K/AKT pathway that phosphorylates and deactivates GSK3β, block BMAL1 and CLOCK from GSK3β-induced phosphorylation and ubiquitination-dependent degradation; 2) To bind with the E-Box sequences associated with BMAL1/CLOCK complex on the promoters of *PER2* and *Rev-Erbα* to negatively regulate the transcriptional activation activity of promoters (Figure 7). As it has been suggested that the expression of about 5%-10% genes in mammal genome are clock-controlled, involving in many processes such as cell metabolism, cell apoptosis, cell cycle and DNA damage response [39, 40]. Thus, by regulating circadian system, the impact of PIWIL2 may expand to various CCG and associating pathways. Taken together, our work described a novel funtion for the cancer/testis antigen PIWIL2 in regulation of the circadian clock, providing a molecular link between spermatogenesis as well as tumorigenesis to the dysfunction of circadian rhythms.

## MATERIALS AND METHODS

### Mices and *in vivo* injection

This study was approved by the Institutional Animal Care and Use Committee at West China Hospital of Sichuan University. All experiments were performed in accordance with relevant guidelines and regulations.

Male C57BL/6 mice (6–8 weeks of age; Experimental Animal Center of West China Hospital, Sichuan University, China) were kept an alternating light/dark (LD) regime with 12 hr of light (lights on at 06:00) and 12 hr of darkness (lights off at 18:00) per day with access to standard chow diet and tap water *ad libitum*.

Briefly, each mouse testical was injected with 30ml of PBS(-) containing 0.6 ml *in vivo* jetPEI (Polyplus-transfection SA, Illkirch, France) and 750 mg shRNA plasmids. As parallel control, non-specific shRNA were injected into the right side testical, while *Piwil2*-specfic shRNA plasmids were injected into the left one of the same mouse. The injection was performed as described previously [41], and anti-MVH was deployed as non-specific control (Supplementary Figure 3). Testis samples were collected 72 hours after injection.

### Plasmids and shRNA

Full-lengthed *PIWIL2* as well as a set of deletion mutants were constructed in our previous study [23]. *Piwil2*-specific shRNA (sh*Piwil2*) was synthesized and cloned into shRNA expression vector pGPU6/GFP/Neo (GenePharma, Shanghai, China). The target sequence of sh*Piwil2*, 5′-CUA UGA GAU UCC UCA ACU ACA GAA G-3′, was based on pervious reports [23, 42].

### Antibodies

The antibodies used in immunofluorescence, IP, ChIP and western blotting experiments were listed below: mouse monoclonal anti-PIWIL2 (Zen Bioscience, Chengdu, China), rabbit polyclonal anti-PIWIL2 (Santa Cruz, Dallas, TX, USA), rabbit polyclonal anti-BMAL1 (Abcam, Cambridge, UK), rabbit polyclonal anti-CLOCK (Cell Signaling Technology (CST), Danvers, MA, USA), mouse monoclonal anti-GSK3β (Zen Bioscience), rabbit monoclonal anti-pGSK3β(S9) (CST), rabbit monoclonal anti-pAKT(S473)(CST), rabbit polyclonal anti-c-SRC (Abcam), mouse polyclonal anti-c-SRC (CST), mouse monoclonal anti-GAPDH (CST), mouse monoclonal anti-HA-tag (CST), rabbit polyclonal anti-PI3K/p85(CST) and rabbit polyclonal anti-Ubiquintin (Abcam).

### Cell culture

Mouse spermatogoniacell line GC-1, mouse spermatocyte cell line GC-2 and cervical cancer cell line HeLa were maintain in State Key Laboratory of Biotherapy of West China Hospital. All cell lines were maintained in RPMI-1640 medium (Thermo Fisher Scientific, Waltham, MA, USA) containing 10% heat-inactivated FBS, 100U/ml penicillin and 100μg/ml streptomycin, on 25 cm^2^ culture dishes in a humidified atmosphere containing 5% CO2 incubator at 37°C. The transfection was performed with JetPRIME (Polyplus-transfection SA) according to the manufacturer's protocol. For stable knockdown cell lines, transfectants were selected by being cultured in medium containing predetermined concentration of G418 (Solarbio, Shanghai, China) for at least one month. For treatment of kinase inhibitor, cells were pretreated with 10 μM TWS119 for 6 hrs, 50 μM LY294002 for 4 hrs, 1 μM Saracatinib for 4 hrs or 1 μM Bosutinib for 4 hrs, respectively.

### Immunofluorescence

For fluorescent immunohistochemistry (IHC) analysis, tissues were formalin-fixed, paraffin-embedded and sectioned at a thickness of 4 um. The slides were dried at 60°C for 1h, deparaffinized at 75°C for 4 min, incubated overnight at 4°C with primary antibody, and finally incubated with fluorescent-labeled secondary antibody for 1 h at room temperature.

For fixed cell immunofluorescence, cells were fixed with 4% paraformaldehyde in PBS for 15 min and permeabilized with 0.5% Triton X-100 for 10 min, blocked with 1% bovine serum albumin (BSA) for 30 min, incubated overnight at 4°C with primary antibody, and finally incubated with fluorescent-labeled secondary antibody for 1 h at room temperature. Each step was followed by 5-min washes in PBS twice.

The prepared specimens were counterstained with 5 μg/mlDAPI for 2 min and observed with a confocal microscope (Olympus, Tokyo, Japan).

### Western blotting and immunoprecipitation (IP)

Harvested cells were lysed in ice-cold universal protein extraction buffer (Bioteke, Beijing, China) supplemented with protease inhibitor cocktail (Roche, Basel, Switzerland) for 30 min. Cell lysates were separated on SDS-page gel and transferred to PVDF membrane (Millipore, Billerica, MA, USA). Membranes were blocked in TBS-T Buffer (50 mM Tris-HCl, 150 mM NaCl, 0.1% Tween, PH7.6) supplemented with 5% nonfat dry milk, incubated overnight at 4°C with primary antibodies followed by 5 min washes in TBS-T for three times and incubated with HRP-labeled secondary antibody for 1 hour at RT. Specific proteins were visualized using enhanced chemiluminescent HRP substrate (Millipore). For phosphorylated protein detection, cell lysates were separated on Phos-tag Agarose SDS-PAGE gel (Boppard, Beijing, China) according to the manufacturer's protocol.

For IP assay, cell lysates prepared from 1 × 10^7^ cells were incubated with 0.8μg target antibody for 2 hours at 4°C with gentle inverting (equivalent cell lysates were incubated with IgG as input control), then incubated with 20 μl of protein G&A agarose (Beyotime) overnight, and precipitated by centrifugation at 12 000 g for 1 min. Complexes were washed four times in ice-cold PBS Buffer (pH7.4), and subjected to western blotting.

### Chromatin immunoprecipitation (ChIP) and real-time PCR

ChIP assay was performed using a ChIP Assay Kit (Beyotime) according to the manufacturer's directions. Cells were formalin-fixed for 10 min, then crosslinking was stopped by adding 125 mM glycine. Lysed with SDS lysis buffer containing protease inhibitor cocktail (Roche), cell lysates were ultra-sonicated by an Ultrasonic cell lyser, and immunoprecipitated with 2 μg antibodies. Eluted and purified DNA was subjected to PCR using primers as follows: Per2 sense: 5′-ATG TAA AAA GAG CGA CGG GC-3′; Per2 antisense: 5′-AGC AGC CCA AGG AAC TTC C-3′; Rev-erb sense: 5′-CTC GTT ACA TAA TGA GCT CC-3′; Rev-erb antisense: 5′-CAG GAA TGG CTC CAT GTT AC-3′.

Quantitative PCR were performed in an iCycler IQ real-time PCR Detection System (BioRad, Hercules, CA, USA), with a first denaturation step at 94°C for 10 min, followed by 45 cycles comprising denaturation at 94°C for 20s, annealing at 58°C for 30s and extension at 72°C for 40s. Inputs removed before applying antibody were deployed to normalize for differences in the amount of DNA in each PCR.

### Electric mobility shift assay (EMSA)

EMSA was carried out using a Chemiluminescent EMSA Kit (Beyotime) according to the manufacturer's directions. Biotin-labeled probes were synthesized (BGI) as follows: Per2 E-box: 5′-CGG GGG CGG GCG CGG CGC GCG CGG TCA CGT TTT CCA CTA TGT GAC AGC GGC GA-3′; Per2 E-box mutated: 5′-CGG GGG CGG GCG CGG CGC GCG CGG T*CG CGC G*TT CCA CTA TGT GAC AGC GGC GA-3′; Rev-erb E-box: 5′-CGA GGC GCT CCC TGG GAT CAC ATG GTA CCT GCT CCA GTG CCG-3′; Rev-erb E-box mutated: 5′-CGA GGC GCT CCC TGG GAT *CGC GCG* GTA CCT GCT CCA GTG CCG-3′. Nuclear exacts were prepared using a Nuclear and Cytoplasmic Protein Extraction Kit (Beyotime) according to the manufacturer's directions.

### Dual-Luciferase assay

For luciferase analysis, sequences of E-Box region and flanking 6 bp at each side were cloned into pGL3 reporter vector. 100 ng plasmid DNA and 100 ng renilla control plasmid were co-transfected into HeLa cells. Dual luciferase-activity assays were performed 48 hours after transfection according to the manufacturer's directions (Promega, Madison, WI, USA).

### Statistical analysis

All experiments were repeated at least three times unless stated otherwise. Western blot results were quantified using ImageJ software. To determine the rhythmically express of proteins, JTK_Cycle software were employed as previously described [43]. Differences between experimental groups were determined using Student's *t* test. Statistical significance was accepted when *P* < 0.05.

## SUPPLEMENTARY MATERIALS FIGURES


