# Supplementary material for: Cancer/testis antigen PIWIL2 suppresses circadian rhythms by regulating the stability and activity of BMAL1 and CLOCK
Source: Oncotarget. 2017 Jul 4;8(33):54913–24. doi: 10.18632/oncotarget.18973 (PMC5589630; doi:10.18632/oncotarget.18973)
Supplement: Supplementary file 1 [file oncotarget-08-54913-s001.pdf]

Cancer/testis antigen PIWIL2 suppresses circadian rhythms by regulating the stability and activity of BMAL1 and CLOCK

SUPPLEMENTARY MATERIALS

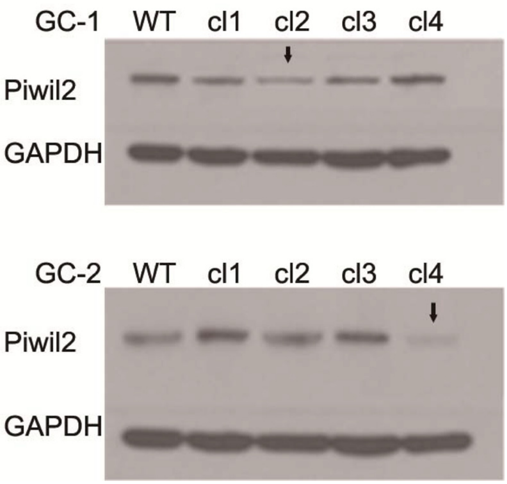

Supplementary Figure 1: Establishment of Piwil2 knocked-down stable cell lines. GC-1 and GC-2 cells were transfected with shPiwil2 plasmids and then selected with Hygromycin B (0.250 mg/ml for GC-1 and 0.350 mg/ml for GC-2, respectively). Hygromycin B resistant clones were appraised with western blotting analysis with anti-Piwil2 antibody. Black arrows indicate clones that expressed the lowest level of Piwil2 and were chosen for followed experiments.

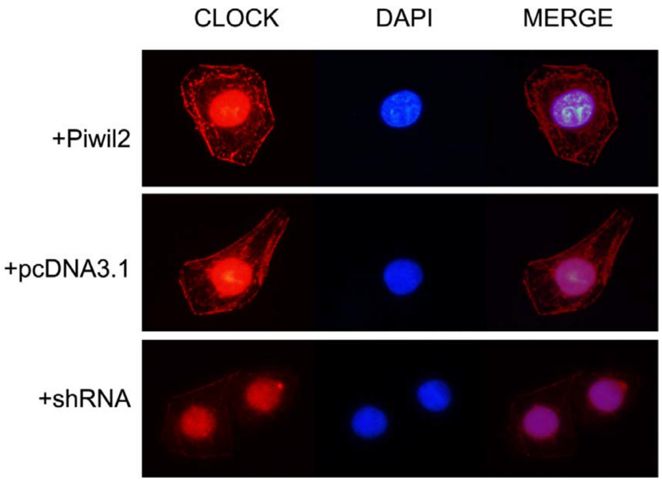

Supplementary Figure 2: Knockdown of PIWIL2 down-regulates CLOCK expression mostly in the cytoplasm.

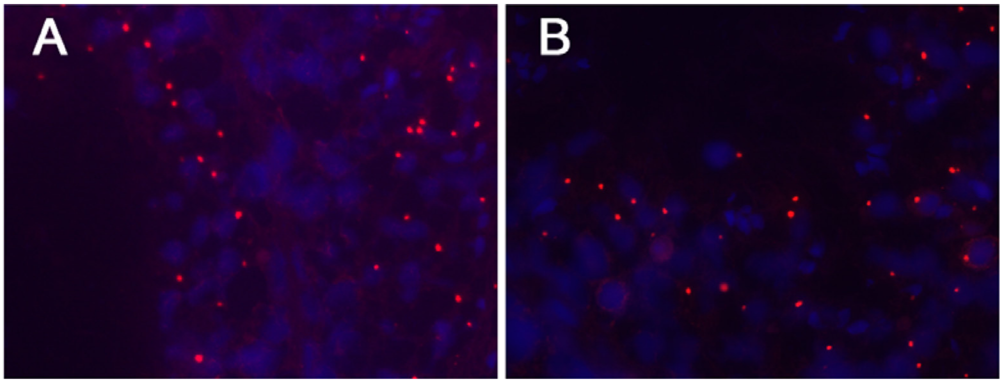

Supplementary Figure 3: Fluorescent immunohistochemistry (IHC) showed expression of MVH in mouse testis injected with *Piwil2*-specific shRNA vectors (**A**) or empty vectors (**B**).
